# On the Artificial Production of Bone

**Published:** 1859-06

**Authors:** Leopold Ollier


### ON THE ARTIFICIAL PRODUCTION OF BONE.

BY DR. LEOPOLD OLLIER.

[Translated from the "Journal de la Physiologie," by E. L. HOLMES, M. D., of Chicago.]

### *Conclusions, and their Application to Surgery.*

1. THE reproduction of bone proceeds from the inner surface of the transplanted periosteum. Wherever a portion of this membrane can be transplanted, new bone is formed, either adherent to the bone from which it is taken, or entirely independent of it, according as it is suffered to remain, partially connected with the rest of the periosteum, or is completely detached from it.

2. In transplanting portions of the periosteum, bone of various forms and dimensions can be obtained, circular, in the form of the figure 8, spiral, etc.; according to the shape and position of the flap, consequently, we have perfect control over the direction and extent of the process of ossification.

3. Bones thus developed are not simple, shapeless concretions of calcareous matter; they consist of true bone, found with all the anatomical characteristics of true bone. In proportion to their development, they resemble normal bony tissue, with an external compact layer, and a medullary cavity in the interior. This cavity is filled with a marrow, resembling, in all respects, that of healthy bones.

4. The new bone is developed in the sub-periosteal blastema, which exists normally upon the inner surface of the periosteum. This proposition is demonstrated by an examination of the formation of the new bone, and by removing this layer of blastema, when the production of bone is suppressed, or at least, for a time arrested.

5. This blastema consists especially of free nucleoli, enclosed in cells, floating in a semi-liquid, transparent, or finely granular material, and mingled with more or less fibrinous elements.

These various embryonic elements develop and multiply in the exudation (at first amosphous,) furnished by the capillaries of the periosteum.

6. The sub-periosteal product, which is observed the first few days following transplantation, is generally cartilaginous; but the succeeding development of bone progresses without this intermediate element. This cartilage, moreover differs in structure and appearance of its anatomical elements, from the normal cartilage of the epiphysis.

7. An analogous membrane is found after a time, upon the surface of bone, from which the periosteum has been removed.

8. When we removed a bone, or a portion of a bone, leaving its periosteum attached to the tissues which normally cover it, we observed at the end of a certain time, that this bone, or portion of bone, is reproduced to a quarter or less extent. The reproduction is sometime perfect. The soft tissues can not supply the place of the periosteum; nor do they directly contribute to the ossification.

The reproduction of bone is in proportion to the amount of periosteum preserved in the wound. When a segment of bone, embracing its whole thickness is removed, its reproduction depends solely upon the periosteum.

9. After the resection of the articular extremeties of two contiguous bones, a new articulation is formed, if the capsule and ligaments are left on all sides, connected with the periosteum of the resected bones. The two bony extremeties are remodeled independently.

The surgical deductions, which we may draw from our experiments, are numerous; but we will only mention the most important, which refer to resections, and to autoplastic operations.

In resections, art should not be content simply to remove the diseased portions; it should also endeavor to reproduce the fragments of bone which it has destroyed. Experimental physiology suggests to us the means; it teaches us why resections have hitherto been so seldom followed with reproduction of bone, and shows what course we should follow in the future. The preservation of the periosteum is of the highest importance. We have endeavored to demonstrate in a recent work, that, in spite of certain inherent anatomical and physiological difficulties, this is practicable, partially at least; and we have proved that clinical observation has already fully confirmed the truth of the assertion. As regards autoplasty, we think that the continuation of ossifying secretions from the wide surface of the transplanted periosteum, increases its importance and extends its application. Since, in animals, we obtain bone wherever we transplant periosteum, we can expect similar results, under certain circumstances, in man, by obtaining portions of the periosteum within the flaps. Innumerable difficulties arise in our mind, when we reflect upon the realization of this idea, which we should see in order to avoid them. But they do not seem wholly unsurmountable.

Periosteal astoplasty (operations, whose object is to reproduce bone by means of transplanted periosteum,) is now a rational undertaking.

There is still another application of these principles not directly illustrated by experiments just reported, but yet in a measure related to them, which we have verified in especial experiments; we refer to the rational mode of diminishing the chance of suppurative inflammation of the bones, after amputations, and of inducing thereby union of the flaps by first intention. The periosteum unites with bony more readily than with any other tissue. The nature of the organizing elements which cover the inner surface of the membrane, and its especial function in ossification, enable us readily to comprehend the causes of this result, which experiment also demonstrated.

We believe that it will prove very useful after amputation, to cover the end of the bone and medullary canal with a small piece of periosteum.

Experiments upon animals illustrated the feasibility of this plan already proposed, but too often neglected, as it seems to us, without sufficient cause. The reasons which lead us to recommend it now, are not those formerly given; they are based upon physiological facts. The difficulty of the operation ought not to influence us, when so much advantage is at stake. We need fear no accident; at most, this step in the operation may prove simply useless.—*Chicago Med. Jour.*

